# Combined Training of One Cognitive and One Metacognitive Strategy Improves Academic Writing Skills

**DOI:** 10.3389/fpsyg.2016.00187

**Published:** 2016-02-23

**Authors:** Anke Wischgoll

**Affiliations:** Department of Psychology, Educational and Developmental Psychology, University of FreiburgFreiburg, Germany

**Keywords:** academic writing skills, combination of strategies, self-monitoring, summarization, text structure

## Abstract

Academic writing is a challenging task. Expert writers apply various writing skills as they anticipate the reader’s view of their text while paying attention to structure and content. Research in the high school setting shows that the acquisition of writing skills can be supported by single-strategy training. However, research in higher education is scarce. We tested whether the development of academic writing skills can also be effectively supported by training single strategies or even combined strategies. As metacognition is an important skill for advanced and adult learners, we focused in this study on the benefit of combined cognitive strategies with and without a metacognitive strategy. An experiment including three conditions was conducted (*N* = 60 German-speaking psychology undergraduates, *M* = 22.8, *SD* = 4.4), which lasted for three hours. Each group received a modeling intervention of a basic cognitive strategy on the application of text structure knowledge. Two groups received an additional modeling intervention with either a cognitive strategy treatment on text summarization or a metacognitive strategy treatment on self-monitoring the writing process. One group received no further strategy treatment. Prior knowledge and learning outcomes were measured with a specially developed test on academic writing skills. In addition, all participants wrote an abstract of an empirical article. We found that learners who received the additional self-monitoring strategy intervention benefited significantly more in terms of acquisition of academic writing skills and the quality of their texts than learners who did not receive this intervention. Thus, the results underline the importance of self-monitoring strategies in academic writing. Implications and further research opportunities are discussed.

## Introduction

Writing is important. It can be used both as a tool for learning and as a tool to convince others of the writer’s argument (Graham et al., 2013). Accordingly, it is a prerequisite for academic life with respect to preparing and publishing research. Writing a text requires recursively rewriting it to come at the writing goal (i.e., learning goal or communication goal; Rijlaarsdam et al., 2004). Thereby, cognitive and metacognitive processes are involved (Flavell, 1976; Flower and Hayes, 1984; Bereiter and Scardamalia, 1987; Flower, 1998; Graham et al., 2005; Graham and Perin, 2007). Beginning academic writers often struggle when balancing cognitive and metacognitive processes. Writing research has shown that the use of cognitive and metacognitive strategies can strongly influence writing quality. Cognitive strategies, such as planning, translating and reviewing, are directly related to the writing process; metacognitive strategies facilitate monitoring the writing process and deciding how to align cognitive strategies in order to reach the intended writing product (Hayes and Flower, 1980; Flavell, 1987; Hayes, 2012).

The training of academic writing skills is essential for scientists at the start of their career. A particular challenge inherent in academic writing is to take into account the reader’s view (Kellogg, 2008). Mindful of the target audience, academic writers apply elaborated strategies to both structural and content problems (Hayes et al., 1987). In a recent review, Schriver (2012) described the application of genre knowledge, the arrangement of non-related text parts into a coherent whole, and balancing the appropriate dose of information between content and target audience in a community-specific manner as main skills in professional communication, e.g., academic writing. This notion is consistent with Spivey’s (1990) description of skills needed for academic writing: selecting, organizing and connecting information.

Activities such as instruction on text structure, text summarization strategies, and self-regulation strategies seem to be helpful to fulfill these requirements, and have proven to be successful in improving writing quality in high schools (Graham and Perin, 2007; Graham et al., 2012). Although these activities are recommended for higher education too, studies on their effectiveness in this setting are scarce. However, a very recent study (MacArthur et al., 2015) on strategy instruction in writing training for college students showed promising findings concerning the effectiveness on writing quality. In the present study, we tested the effectiveness of strategy training interventions which are recommended for higher education, and analyzed whether academic writing skills can be effectively supported by teaching a combination of these strategies. More specifically, we assumed that (1) the training of a text structure knowledge application strategy together with a self-monitoring strategy benefits the acquisition of academic writing skills more than training of the former strategy alone or in combination with a summarization strategy, and (2) the training of a text structure knowledge application strategy combined with a self-monitoring strategy benefits the text quality more than training of the former strategy alone or in combination with a summarization strategy.

## Theoretical Background

From the developmental perspective, writers pass through three stages from novice to expert writer. At the novice stage, writing is used to tell what one knows, at the intermediate stage it is used to transform the text to the author’s benefit, and at the final, expert, level, it is used to craft the text mainly for the reader’s benefit (Hayes and Flower, 1980; Bereiter and Scardamalia, 1987; Kellogg, 2008). The more advanced the writer is, the more important the prospective assumptions regarding the reader’s understanding of the text become (Kellogg, 2008).

Writing, and particularly academic writing, is a complex process. Hayes and Flower (1980) identified that expert writers do not apply individual skills, but rather a combination of skills in their writing process. The authors described how experts organize their writing process and which influences on the writing process they take into account: While writing, writers pass through, in a recursive manner, the processes of planning, translating, and reviewing; as external influences, writers take into account the task environment (e.g., topic, audience, text produced); and as an internal precondition, they determine their own prior knowledge about and experience with the writing topic, the audience and their writing skills (Hayes and Flower, 1980; Hayes, 2012). The before mentioned processes are self-regulatory processes including goal setting, organizing information, evaluating, and adaptive revisions. The recursive sequencing of these writing processes is attributed to cognitive monitoring (Hayes and Flower, 1980; Zimmerman and Kitsantas, 2007). Among the various self-regulations skills, self-monitoring is noted to be promising for learning to write (Cresswell, 2000). It enables learners to get information about task related cognitive and affective processes, fosters memory retrieval and evaluation of the gained information (Reder and Schunn, 1996).

What can we learn from expert writers? They manage the writing process best by monitoring the planning, translating and reviewing processes of the written product (Hayes and Flower, 1980; Hayes, 2012). To monitor the writing process, expert writers keep in mind what they want to tell, and consider what they have already written. Furthermore, they take into account the reader’s perspective. In other words, they maintain a balance between retrospectively and anticipatorily judging the written text from their own and from the reader’s presumed perspective (Hayes and Flower, 1980; Kellogg, 2008; Kellogg and Whiteford, 2009; Hayes, 2012). The execution of all aspects of the writing process places a heavy burden on the working memory (Hayes and Flower, 1980; Kellogg, 1996; McCutchen, 1996; Hayes, 2012), and to relieve this burden, expert writers should seek to automatize skills that support the writing process (McCutchen, 1996; Kellogg et al., 2013). For this reason, writing skills require practice (Hayes and Flower, 1980; Bereiter and Scardamalia, 1987; Kellogg, 1996; Kellogg and Whiteford, 2009; MacArthur et al., 2015). Writing skills which are acquired through practice may then also be applied in combination.

Undergraduates are expected to be (advanced) knowledge transformers (Bereiter and Scardamalia, 1987; Kellogg and Whiteford, 2009). In higher education, the foundation is laid for becoming an expert writer, reaching the stage of knowledge crafting. This should focus on two aspects. First, writing skills, strategies, and knowledge relevant to academic writers have to be trained. Second, this training must be organized in a way that takes into account the limited capacity of the working memory and the need for practice. The concept of “observing and doing” seems to be a promising method for acquiring academic writing skills (Kellogg, 2008).

### Learning by Observing and Doing

To train writing skills, Kellogg (2008) recommends both learning by observing and learning by doing. He claims that these two training methods complement each other if they are administered in appropriate proportions.

Learning to write by observing is an often practiced method (Rosenthal and Zimmerman, 1978; Bandura, 1986, 1997; Schunk, 1987, 1991). It can take place by observing a mastery model or a coping model. Mastery models perform the task as they adapt the task-relevant knowledge, skills, and strategies, without struggling. Coping models are less competent; they struggle and deal with the challenges. In high school students, Braaksma et al. (2002) were able to show that similarity between model and learner in terms of competence was conducive for learning to write: Weak learners improved by observing non-competent models, while better learners improved by observing competent models (Braaksma et al., 2002). The results were refined in a further study (Braaksma et al., 2006), in which learners reported cognitive and metacognitive activities such as observing, comparing, evaluating, and reflecting on activities while they were observing the model. These activities are known to foster learning to write. Zimmerman and Kitsantas (2002) tested the modeling effect in academic writing with undergraduates, who observed either a mastery model performing the writing task perfectly or a coping model gradually improving his/her writing skills. They found that learning by observing any type of model can lead to better writing quality than learning without observing a model, but that the coping model was the most effective.

Learning to write by doing follows on from observational learning. The model of cognitive apprenticeship (Collins et al., 1989) and the socio-cognitive model (Schunk and Zimmerman, 1997) represent sequences for learning by first observing and then doing. Kellogg (2008) recommends the model of cognitive apprenticeship for the development of writing skills because it combines observational learning and practice with gradually fading support. It is described from the expert’s perspective, with experts supporting novices through modeling, coaching, scaffolding, and fading. In the model by Schunk and Zimmerman (1997), the four phases which are postulated for the development of writing skills are described from the learner’s perspective: First, the learner observes the model (*observation*); second, the learner emulates the model’s behavior (the learner does not merely copy what the model did but develops it further) (*emulation*); third, the learner internalizes emulated skills (*self*-*control*); and finally, the learner’s skills become more flexible through working on transfer tasks (*self*-*regulation*). In the emulation phase, feedback encourages the learner to enhance his/her performance and to develop self-regulatory guidelines. In the self-regulation phase, self-efficacy and intrinsic motivation promote learning. A tried and tested strategy instruction model for writing development is described by the Self-Regulated Strategy Development (SRSD) framework (Graham, 2006), which consists of six steps that can be thoughtfully combined and modified: (1) develop background knowledge, (2) discuss it, (3) model it, (4) memorize it, (5) support it (gradually fading), and (6) independent performance (Harris and Graham, in press). This model also describes learning as a sequence, from modeling, through practicing with fading support, to independent performance. A vast number of studies using the SRSD framework have demonstrated the effectiveness of observing and doing for learning to write in the high school setting (Harris and Graham, in press).

As mentioned above, academic writing is a complex process that requires a variety of skills. On the one hand, writers need to organize their writing as they structure their text and the information they have gained from text sources. On the other hand, they need to evaluate text sources and their own text; they then elaborate their text in recursive loops. Thus, writers apply cognitive and metacognitive strategies in writing. In order to relieve working memory, deliberate application of cognitive and metacognitive strategies frees up capacity for a detailed elaboration of the text.

### Fostering Writing Skills

Research on writing at the school level is well established and provides practicable implications for successful classroom instruction. Meta-analyses of writing instruction (Hillocks, 1986; Graham, 2006; Graham and Perin, 2007; Graham et al., 2012) have validated different forms of training which support the improvement of students’ writing quality. They suggest teaching writing skills, strategies, and knowledge directly (for an overview see Graham et al., 2013). In a meta-analysis of writing instruction for adolescent students, Graham and Perin (2007) showed that a number of cognitive and metacognitive strategies can support the acquisition of writing skills. Cognitive writing strategies can support the process of organization while writing. In this respect, writers identify main ideas and their relations within the text source and to their own writing; organizational strategies can also refer to structuring text content (Weinstein and Mayer, 1986). Metacognitive writing strategies can support the process of control and management of the writing process, or more precisely the application of cognitive strategies in the writing process (Flavell, 1976). Graham and Perin (2007) found that the training of organizational strategies such as summarization (e.g., Chang et al., 2002) and text structure application (e.g., Fitzgerald and Teasley, 1986) was highly effective for fostering writing quality. Furthermore, the training of self-regulation strategies such as reflection or monitoring (e.g., Bryson and Scardamalia, 1996) has also been shown to be highly effective for promoting stronger writing quality.

Compared to research on writing skills in high schools, respective research in higher education is rare. Kellogg and Whiteford (2009) believe that the recommended training interventions for high school students are also suitable for college students. They name four effective interventions which can be seen as preparatory for writing and ease temporarily the demands of the task: prewriting activities, inquiry activities for content developing, collaborating with peers, and explicitly goal setting. Another three interventions, that is strategies for planning, revising, and/or editing, sentence combining, and summarization - also rated as promising – concern the process of writing itself.

Which training units seem to be helpful for basic training in academic writing? As academic writing includes building a macrostructure of the whole text as a first step, training on text structure is recommended (Kellogg and Whiteford, 2009; Graham et al., 2012). In order to elaborate the microstructure of the text as a second step, both the summarization strategy and the self-regulation strategy may be highly effective for undergraduates. Summarization training may help novice academic writers to learn to select the main statements from source texts for their own text, while keeping in mind the intention behind their writing, and to translate these main statements into a concise language (Kellogg and Whiteford, 2009). Self-regulation training may help them to become more aware of the execution of their writing process and of their addressee’s possible mental representation of the text (Kellogg and Whiteford, 2009).

### What is Crucial for Academic Writing?

Based on the aforementioned theoretical background, we can derive the following task analysis: writing an academic text is an alternating process of preparation and generation. During preparation, the author selects information concerning the main proposition of the text from source text. As academic texts, and especially empirical articles, are all structured in the same way, knowing the text structure helps the author to find relevant information more easily. Text structure knowledge also supports the generation of one’s own text. While writing his or her own text, the author assigns information to text sections, and while reading his or her own text, the author checks the text regarding whether the information is rendered completely and correctly, and regarding whether the text is arranged in accordance with the reader’s anticipated understanding; this includes, besides language proficiency, text coherence and awareness of the reader’s understanding. While writing, the author benefits from text structure knowledge as he or she is able to assign information to the text while considering the text structure. Writing a new text referring to multiple sources requires reducing information, carving out essential statements, and shaping the information and statements to correspond to the text structure. Writing also requires the author to check over his or her own actions. The author monitors how information is assigned and arranged, and checks for coherence and readability of the text (see **Table 1** for an overview).

**Table 1:** Single- and combined-strategy treatments with their expected learning outcome.

	Writing process	Basic control group	Combined control group (summarization)	Experimental group (self-monitoring)
Basic training	Preparation	Text structure knowledge application strategy: Finding information in certain text sections		
	Generation	Text structure knowledge application strategy: Assigning information to text sections		
Additional training	Preparation		Summarization strategy: Reducing information	Self-monitoring strategy: Checking the selected text passages
		
	Generation		Summarization Strategy: Carving out the main aspects of the text	Self-monitoring strategy Checking the writing process (structure, message, audience)
		
Effect of skill on		Assignment of information		
		
			Selection of information	Arrangement of information
		
Expected learning outcome		Text structure knowledge		
		
			Text reduction	Application of text structure knowledge
		
Expected abstract quality		Well-structured		
		
			Well-selected and arranged information	Easy to read

This task analysis identified the main aspects which need to be taken into account and related to one another in order to master an academic writing process: structure, content, and coherence/readability. Hence, the current contribution focuses on strategies which can be applied globally across the whole writing process: application of text structure knowledge, text summarization, and self-monitoring of the writing process.

#### Application of Text Structure Knowledge

In the case of academic writing, different text genres exist. In psychology and in educational sciences in general, the empirical article is assumed as the key genre for national and international discourse (Swales, 1990). This genre is a well-accepted means to transmit research-relevant information to the research community in a concise but elaborated style. On the one hand, the clear structure of the text helps readers to easily find what they are seeking; on the other hand, the text structure helps the writer to coordinate ideas and intention (Goldman and Bisanz, 2002). Englert (2009) confirmed the importance of text structure knowledge as training for the writer to organize the writing process.

#### Text Summarization

In empirical articles, information from other texts is typically reproduced, particularly in the introduction section. Expert writers select such information from text sources. With this information, they form a new text with derived, new information. For this purpose, they delete unimportant and redundant information, generalize connected propositions, and construct topic sentences out of a joint set of propositions. Deletion, generalization, and construction are macrorules which help to reduce and organize information (Van Dijk and Kintsch, 1983). In a study about paraphrasing expository texts, it was found that junior college students were able to delete redundant information but displayed significant deficits in generalization and construction (Brown and Day, 1983). A study in which writers wrote summaries for themselves or for readers found developmental differences between novice and advanced writers (Hidi and Anderson, 1986): when writers wrote summaries for themselves as a preliminary stage to writing summaries for others, it was found that novice writers summarized their texts mainly through deletion and copying. In contrast, advanced writers summarized in a constructive way, by emphasizing an intended message of the text for their own writing. In a recent study on summary writing performance with undergraduates, Li (2014) found that summarization for well-structured text genres fits well with writing quality.

#### Self-Monitoring

Self-regulation skills such as self-monitoring are prerequisites for writing well in higher education (Cho et al., 2010). Self-monitoring can be distinguished into formal and informal activity. Informal self-monitoring helps coping with less complex or familiar tasks in a (semi-) automated way; by contrast formal self-monitoring suits for comprehending new reading material and acquiring new skills as it involves systematic planning and controlling (Zimmerman and Paulsen, 1995). As the research presented in this article concerns the complex process of writing, the term self-monitoring is used in the sense of a formal activity.

Self-monitoring of a learning process, such as learning-to-write, implies that learners systematically observe and evaluate their learning while they are still involved in the current learning activity (Zimmerman and Paulsen, 1995; Zimmerman, 1998). In this way learners can identify discrepancies between their intended product and the current state of the product (Butler and Winne, 1995). As self-monitoring provides internal feedback on the quality of the current learning process, learners can adapt their cognitive strategies.

Zimmerman and Kitsantas (2007) could show that product oriented self-monitoring can be effective. Learners who were asked to monitor their progress according to the intended product performed better than students without a product oriented monitoring-prompt. Cho et al. (2010) defined self-monitoring as involving the evaluation of the written text, the targeted message of the text, and the mismatch between the two. In their study, they investigated the development of self-monitoring skills through self-evaluation and peer feedback and the correlation with writing quality. As a main result, they found that students with well-developed self-monitoring skills enhanced their writing quality significantly. This finding is in line with findings by Harris et al. (1994), who distinguished the self-monitoring process into performance monitoring (with a focus on task performance) and attention monitoring (with a focus on the on-task behavior). In their study, they found that achievement scores were higher in the performance monitoring condition than in the attention monitoring condition (Reid and Harris, 1993). These results were replicated in a further study (Harris et al., 1994). Altogether, these studies clearly indicate that the self-monitoring of a writing task, that is, the writer’s evaluation of the difference between written product and writing goal, is essential for improving writing quality.

#### Combination

In a meta-analysis, Graham and Perin (2007) raised the question of whether writing quality could be developed even more effectively by applying two or more activities in combination. Earlier studies provided first evidence that the combination of process writing and strategy instruction can indeed be beneficial (e.g., Danoff et al., 1993). However, information on how teaching a combination of strategies affects the improvement of writing quality is still lacking. A recent study (Reynolds and Perin, 2009) compared two techniques for teaching writing: text structure instruction and summarization strategy, with the former focusing on text characteristics and the latter focusing, among other things, on note-taking and composing. The results showed that neither strategy was generally superior to the other, but that each brought with it benefits in a different area. Text structure instruction enhanced writing quality and content knowledge, while the summarization strategy enhanced the writer’s ability to find and include new ideas in the text. These findings indicate that the combination of strategies can maximize writing quality.

### The Present Study

The aim of our study was to analyze whether the acquisition of academic writing skills can be effectively supported by teaching a combination of cognitive and metacognitive strategies. We selected the genre of the empirical article as it is a prominent representative for academic texts (Swales, 1990). We assumed that text structure knowledge is a prerequisite for writing an empirical article. A strategy to apply text structure knowledge helps writers to find relevant information in empirical articles concerning their current research interests (Meyer and Poon, 2001). Moreover, such a strategy helps writers to compose their text, as they are able to assign certain contents to the corresponding sections of their empirical article. In higher education, writers need to anticipate the potential view and understanding of the reader. With the aim of testing their efficacy for academic writing, we selected two promising strategies which were found to be highly effective for improving writing quality, the text summarization strategy and the self-monitoring strategy (Harris et al., 1994; Graham and Perin, 2007). The training of the *text structure knowledge application* strategy served to build up a shared foundation for academic writing in the genre empirical article. Subsequently, we expected that a *text summarization* strategy (selecting paragraphs, sentences, and keywords by taking into account the intended message of the text to be written) would yield additional value in terms of writers being able to easily select relevant information from the text source and to carve out the main aspects of the text in a concise manner. Following this, a *self-monitoring* strategy (self-evaluating, before and while writing, the (lack of) correspondence between the written product and the writing goal concerning the text structure, the message of the text, and the audience) was applied, which we expected to enable writers to control their selection of text passages and the readability of their text. Moreover, we expected that the self-monitoring strategy would benefit the transfer of text structure knowledge, such as detecting a missing section of an abstract. As Veenman et al. (2004) argued, the more experienced the learners are, the more important metacognitive skills become for mastering cognitive challenges such as academic writing. We propose that the additional training of the self-monitoring strategy will provide benefit in terms of the *acquisition of academic writing skills* and *abstract writing*.

We tested the following hypotheses

Hypothesis 1:

Training to apply text structure knowledge during writing in combination with a metacognitive strategy (i.e., self-monitoring) fosters the *acquisition of academic writing skills* more than training to apply text structure knowledge during writing alone or in combination with a cognitive strategy (i.e., summarization). We assumed that third variables, i.e., *prior knowledge on reproduction of text structure knowledge, prior knowledge of application of text structure knowledge*, and *prior knowledge of reduction of text content*, are related to the *acquisition of academic writing skills*.

Hypothesis 2:

Training to apply text structure knowledge during writing in combination with a metacognitive strategy (i.e., self-monitoring) fosters the *text quality* more than training to apply text structure knowledge during writing alone or in combination with a cognitive strategy (i.e., summarization). We assumed that third variables, i.e., *text quality after summarization* and *text quality after revision*, are related to the *text quality of the abstract*.

## Materials and Methods

The present study was conducted in accordance with the ethical principles of the German Psychological Society [DGPs] (DGP, 2005) and the American Psychological Association [APA] (2010). Guidelines provided by these institutions state that formal informed consent is not necessary when no potential harm or distress is to be expected and/or when normal educational practices are followed as a goal of the research. Prior to their participation, the participants of the present study were informed of the research, duration, and procedures. Participation was voluntary, and it was possible to withdraw participation at any time. All participants provided verbal informed consent prior to data collection. All data were collected and analyzed anonymously.

### Participants and Design

Data were analyzed from 60 German-speaking undergraduate psychology students (female = 48, male = 12) who volunteered to participate in the study. All students were in their first (*n* = 52) or second year (*n* = 8) at the University of Freiburg in Germany (*M* = 22.8 years, *SD* = 4.4). Students were randomly assigned to one of the three conditions of our experimental pre–post-test intervention study: All groups received the same instruction on text structure. In addition, the experimental group (*N* = 20) received prompts for self-monitoring strategies directed at the writing process. One control group (*N* = 20) received no further support, while the second control group (*N* = 20) received an instruction on text summarization as an “add-on”.

### Procedures

The experiment was conducted in a 3-h session including a short break. All participants managed their time individually in a computer-based learning environment without interaction with other participants. We randomly assigned the participants in equal numbers to one of the three treatment conditions: text structure knowledge application strategy only, additional summarization strategy, additional self-monitoring strategy. Participants were not informed about the nature of their condition.

The experiment consisted of two phases: modeling phase and deliberate practice phase. Before the modeling phase, demographic data and self-reported prior knowledge about text structure were assessed. The participants were then tested on their prior knowledge on academic writing using a short version of a specially developed academic writing scale, as well as on current motivation (Vollmeyer and Rheinberg, 2006) and on self-efficacy (Bandura, 2006). Following the modeling phase, the participants were retested on self-efficacy. Following the deliberate practice phase, the participants were tested with the full version of the specially developed academic writing scale.

During the *modeling phase*, the participants of all three groups received training through learning journals, which they read at their own pace. In the learning journal, a peer model – her name was Corinna – illustrated and exemplified her own experience of writing a Bachelor thesis, which took the form of an empirical article. The peer model demonstrated aspects of struggling and offered strategies to master the writing process effectively. All groups read a basic learning journal about the *text structure knowledge application strategy*, i.e., how an empirical text is structured and how one can apply this text structure knowledge to write an empirical article or an abstract. The peer model described the literature search and the writing process as a recursive, interconnected circle. Each text section was addressed in view of the questions arising in the writing process and during the literature search (e.g., Where can I find the answer to my question?) and with respect to the content of an empirical article (e.g., What do I have to assign to each specific text section?). Both additional treatments were presented again as learning journals and focused on writing coherent abstracts taking into account the text structure. In one learning journal, the emphasis was on the *summarization strategy*, with the peer model demonstrating a logical reduction and rearrangement of the text content. The peer model described the relevant steps from macrostructure (e.g., I gain an impression of the subject and the message of the text) to microstructure (e.g., I mark sections for which I think I can write a quick summary) in order to extract the best information from text sources (e.g., I am aware of the information that I want to convey in the text) and arrange the information into a new text (e.g., I check the structure and the logic of the abstract). In the other learning journal, the emphasis was on the *self-monitoring strategy*, with the peer model demonstrating awareness of the reader’s perspective. The peer model described the preparation of the writing process (e.g., Can I create a coherent text from my text selection?; Do I manage to arrange my text so that it is understandable?) and the implementation of the writing process (e.g., Does my writing reproduce the message of the text source?; Can I comprehend my text if I read it from the reader’s perspective?). In this phase, the participants were not allowed to take their own notes.

After the modeling phase, the participants had a 3-min break remaining at their seats. They were requested to do nothing during this break. The screen showed an image of a landscape, and it was not possible to continue until the break was over.

In the deliberate practice phase, the participants were asked to write an abstract of an empirical article. The participants were guided by prompt cards to consider the strategies modeled with the learning journals. For each learning journal, the participants used a single prompt card, on which the main points of the strategy use were presented. For the deliberate practice phase, the participants were given the empirical article as a paper handout. The Sections “Theoretical Background,” “Materials and Methods,” “Results,” and “Discussion” of an original empirical article were each reduced to around 400 words. Title, abstract and keywords were omitted. In the computer-based learning environment, each section was presented separately. The first step was to produce a summary of the article. All participants read each text section and were asked to summarize each section in 40 words or less. They were then told that they would be writing a coherent abstract for this empirical article and that they should keep this in mind during the next two steps. In the second step, the summarized text sections which the participants wrote in the first step were presented in succession, and the participants were asked to reduce the text once more, this time to 20 words or less. In the third and final step, all summarized text sections from the second step were presented together. The participants were asked to compile, from these summaries, a coherent abstract of no more than 100 words.

### Text

For the deliberate practice phase, an original empirical article was selected according to the following criteria: (1) the topic is of general interest, the participants are expected to be familiar with the topic through the media, but not through their education; (2) the text structure is simple; (3) the statistical analyses are easy to follow; (4) the text is written without too many unfamiliar terms; (5) the text is written in German, which is the native language of the participants. In accordance with these criteria, we selected an article on mindfulness-based cognitive therapy (Hülsebusch and Michalak, 2010). To fit in with the time constraints of the study and the desired cognitive load, the article was shortened and divided into four sections (theoretical background, methods, results, discussion). Each section was reduced to around 400 words. Abstract, keywords, and title were omitted.

### Prompt Cards

In the deliberate practice phase, the participants used prompt cards according to their treatment, each reflecting a different strategy: (1) text structure knowledge application strategy, (2) text summarization strategy, and (3) self-monitoring strategy. In all learning journals, the peer model explained how she coped with the challenge of writing her Bachelor thesis. More precisely, she described what she wanted to do, how she did it, which conclusions she reached, and which tricks she learned. Finally, for each strategy, she offered the following prompts for mastering the writing strategy:

The learning journal *text structure knowledge application strategy* focused on the use of text structure knowledge: (1) How is an empirical article structured?, (2) What are the characteristics of each text section?, (3) How can the characteristics help to assign information to a single text section? Furthermore, conventions of language use in empirical articles and abstracts were described.

The learning journal *text summarization strategy* focused on selecting and assigning text information: (1) What is the main proposition of the text?, (2) Which passages, sentences, and words may represent the main proposition?, (3) Which of the selected passages, sentences, and words should be used to write a coherent text?, (4) How can text structure knowledge help to write a coherent text with the selected passages, sentences and words?

The learning journal *self-monitoring strategy* focused on checking the writing process: (1) Did I select and assign all relevant information corresponding to the text structure?, (2) Does the text represent the author’s intention?, (3) Is the selected information strung together in a rational manner?, (4) Can the reader understand what I want to say?

### Measures

In order to measure how the participants fulfill the requirements of academic writing competence, we developed an academic writing skills test corresponding to the task analysis presented in the theoretical background.

#### Academic Writing Items

We developed 19 items according to the main aspects of academic writing skills. As mentioned in the theoretical background, academic writing skills comprise, among other things, text structure knowledge (factual knowledge), application of text structure knowledge, and reduction of text content with respect to completeness and correctness (both procedural knowledge). The items were assigned to one of three subscales which cover three aspects of academic writing: (1) text structure knowledge as factual knowledge (5 items), (2) application of text structure knowledge (six items), and (3) reduction of text content with respect to completeness and correctness as procedural knowledge (eight items). To test their text structure knowledge, the participants were asked, for example, to correctly arrange text section titles of an empirical article, i.e., Abstract, Theoretical Background, Materials and Methods, Results, and Discussion. To test their skill in the application of text structure knowledge, they were asked to assign typical phrases to text sections such as methods or discussion, and to give reasons for their decision. To test their skill in reducing text content with respect to correctness and completeness, they were asked, for instance, to name four keywords to adequately express the message of a text.

These items were tested in a pilot study (*N* = 5) with regard to the clarity of formulation. We subsequently revised the items taking into account the criticisms expressed by the pilot study participants in the interview after the pilot study. Participants’ written answers were rated as right or wrong. To ensure reliability of the rating system, two raters conducted the rating independently, and a high level of inter-rater agreement was achieved [intraclass correlation coefficient *ICC*(31) > 0.80]. Disagreement was resolved by discussion in all cases. Two experienced researchers who have published and reviewed over many years assigned the 19 items to one of the three contexts (text structure knowledge, application of text structure knowledge, and reduction of text content with respect to completeness and correctness). The interrater reliability was excellent [*ICC*(31) = 0.91] (Fleiss, 2011).

#### Writing Quality

The writing quality was measured three times during the writing process. The first measurement time point was the summarization of the text sections of the presented empirical article, the second was the revision of the summarized text, and the third was the finalization of the intended abstract of the presented text sections of the original empirical article. At each time point, we rated the overall quality on a 6-point scale (1 = *disastrous*, 6 = *very good*) (adapted from (Cho et al., 2006)). The rating of the overall quality of the written text focused on whether the text represents the writer’s awareness of the reader, which is a critical aspect of academic writing. Two project research assistants who were familiar with the affordances of academic writing received about 4 h of training on the quality rating scale. Training included practicing making the respective judgment and discussing six cases. The abstracts were rated independently without awareness of the participant’s experimental condition and identity. To calculate the interrater reliability, 20 abstracts were selected. The intraclass correlation coefficient was *ICC* (31) > 0.80, which suggests an excellent interrater reliability (Fleiss, 2011).

#### Academic Writing Skills Test

The pretest of academic writing skills consisted of 10 items assessing text structure knowledge, application of text structure knowledge, and reduction of text content (with respect to correctness and completeness). As all participants were undergraduates in psychology, mainly freshmen, they had no experience with academic writing. For this reason, we refrained from assessing writing quality as a baseline value. The posttest of academic writing skills consisted of two parts: The first comprised all 19 items which capture text structure knowledge, application of text structure knowledge, and reduction of text content with respect to correctness and completeness as subscales. All items were evaluated as right or wrong. All subscales were equally weighted. The second part consisted of the writing quality of the written abstract. Both parts were equally weighted.

### Additional Measures

#### Prior Knowledge About Text Structure

The participants self-reported their prior knowledge about text structure by two items (item 1: “I know how empirical articles are generally organized.”; item 2: “I recognize the text structure easily.”). The items were not significantly related, *r* = –0.011, *p* = 0.931.

#### Motivation

We used the Questionnaire on Current Motivation (Vollmeyer and Rheinberg, 2006) to measure how motivated the participants are to develop their academic writing competence. The scale consists of four subscales: challenge (Cronbach’s alpha = 0.669), interest (Cronbach’s alpha = 0.706), probability of success (Cronbach’s alpha = 0.198), and anxiety (Cronbach’s alpha = 0.636). We deleted one item of the subscale anxiety, which did not seem to be appropriate to the research content of our study. From the subscales interest and anxiety, one item in each was reformulated to better match the sample and the research content of our study. The participants were asked to estimate their current motivation in relation to their academic writing development. They rated each written description on a 7-point scale from 1 (*not true*) to 7 (*true*). The scale was administered before the modeling phase.

#### Self-Efficacy

We followed the guide for constructing self-efficacy scales (Bandura, 2006) to construct a self-efficacy scale focusing on academic writing. In this regard, we took into account the main aspects of our intervention, i.e., reproduction of text structure knowledge, application of text structure knowledge, and reduction of text content. Participants were asked to rate how certain they are that, for example, they “can find certain information in an empirical article” or “can find a precise and concise title for my Bachelor thesis”. For each written description, they rated their confidence from 0% (*cannot do it at all*) to 100% (*highly certain I can do it*) in 10%-increments. The scale was administered before the modeling phase (Cronbach’s alpha = 0.856) and after the modeling phase (Cronbach’s alpha = 0.911). We used this scale to check the responsiveness to the treatment.

## Results

For all statistical analyses, an alpha level of 0.05 was used. Effect sizes for the ANOVAs were calculated using $\eta_{p}^{2}$. We used the effect size measure η^2^ (0.01 as a small effect, 0.06 as a medium effect, and 0.14 as a strong effect (Cohen, 1988)).

### Pre-Analysis

#### Prior Knowledge About Text Structure

Concerning “knowing the text structure of an empirical article” (item 1) we did not find any differences between the groups, *F*(2,57) = 0.463, *p* > 0.05; this is also true for “recognizing the text structure” (item 2), *F*(2,57) = 0.065, *p* > 0.05. Calculating an MANCOVA we did not find that the items influence the dependent variables *academic writing skills* and *abstract quality* significantly, *V* = 0.009, *F*(2,54) = 0.254, *p* > 0.05 (item 1) and *V* = 0.059, *F*(2,54) = 1.705, *p* > 0.05 (item 2).

#### Academic Writing Skills

**Table 2** shows the means and standard deviations for the pretest and posttest in each condition. In the pretest, no significant differences were found across the conditions, *F*(2,57) = 1.85, *p* > 0.05. The short scales used in the pretest also revealed no significant differences between conditions. This was the case for *reproduction of text structure knowledge, F*(2,57) = 0.56, *p* > 0.05, *reduction of text content, F*(2,57) = 1.20, *p* > 0.05, and *application of text structure knowledge, F*(2,57) = 1.03, *p* > 0.05. The average pretest percentage in the three conditions ranged from 30.6 to 39.3% (see **Table 2**), which reveals that the participants had some knowledge about academic writing, but not a great deal. With respect to the subscales of the pretest, the average scores ranged from 10.0 to 20.0% for *reproduction of text structure knowledge*, 18.0 to 28.0% for *application of text structure knowledge*, and 63.0 to 75.0% for *reduction of text content* with respect to correctness and completeness. These results indicate that the participants had only sparse knowledge about text structure and its application, but quite good knowledge regarding text summarization.

**Table 2:** Means and standard deviations for each condition at posttest.

Dependent variable	Basic structure application intervention CG1 (*n* = 20)		Additional text summarization intervention CG2 (*n* = 20)		Additional self-monitoring intervention EG (*n* = 20)	
	*M*	*SD*	*M*	*SD*	*M*	*SD*
**Academic writing test**						
Pretest %	39.3	18.7	30.6	13.2	37.7	13.0
Posttest %	58.9	11.4	56.7	9.8	68.0	13.5
**Skill: reproduction of text structure knowledge**						
Pretest %	20.0	41.0	10.0	30.8	10.0	30.8
Posttest %	78.0	25.0	77.0	21.8	76.0	19.0
**Skill: reduction of text content**						
Pretest %	73.8	23.6	63.8	28.6	75.0	22.9
Posttest %	61.9	17.5	65.0	15.5	63.8	14.0
**Skill: application of text structure knowledge**						
Pretest %	24.0	23.0	18.0	20.4	28.0	22.9
Posttest %	41.7	18.3	35.8	13.5	48.3	18.7
**Skill: Writing quality of the abstract (max. 6)**						
(1) After summarization	3.45	1.00	3.60	1.00	3.80	1.06
(2) After revision	3.55	0.60	3.50	1.03	4.00	1.08
(3) Abstract	3.70	0.87	3.50	0.76	4.70	1.23

#### Motivation

We calculated a MANCOVA to assess whether there is a difference in motivation between the treatment groups. Using Pillai’s trace, we did not find any significant effect of challenge, interest, probability of success, and anxiety, *V* = 0.049, *F*(2,57) = 0.008, *p* = 0.992.

#### Self-Efficacy

We calculated a dependent *t*-test to assess the responsiveness to the treatment, and found a strong, significant effect regarding the responsiveness to the treatment [*t*(59) = –7.715, *p* < 0.001, *r* = 0.71]. The participants experienced significantly higher self-efficacy after the treatment (*M_post_* = 69.23, *SD_post_* = 11.86) than before (*M_pre_* = 62.23, *SD_pre_* = 12.60). This was true for each treatment group: control group [*M_pre_* = 64.45, *SD_pre_* = 13.53; *M_post_* = 71.00, *SD_post_* = 11.58; *t*(19) = –4.287, *p* < 0.001, *r* = 0.70], summarization group [*M_pre_* = 65.00, *SD_pre_* = 10.34; *M_post_* = 71.40, *SD_post_* = 10.33; *t*(19) = –3.757, *p* = 0.001, *r* = 0.65], and self-monitoring group [*M_pre_* = 60.25, *SD_pre_* = 13.74; *M_post_* = 66.20, *SD_post_* = 13.35; *t*(19) = –6.055, *p* < 0.001, *r* = 0.81]. Calculating an MANCOVA we did not find that self-efficacy influenced the dependent variables *academic writing skills* and *abstract quality* significantly, *V* = 0.006, *F*(2,55) = 0.164, *p* > 0.05.

### Posttest Outcomes

In hypothesis 1, we assumed that additional training of a self-monitoring strategy fosters the *acquisition of academic writing skills* more than additional training of a summarization strategy or no additional training. We furthermore assumed that the third variables, i.e., *prior knowledge on reproduction of text structure knowledge, prior knowledge of application of text structure kno*wledge, and *prior knowledge of reduction of text content*, are related to the acquisition of academic writing skills.

Our results show that there is a significant difference between the three treatment groups concerning the acquisition of academic writing skills, *F*(2,54) = 3.913, *p* = 0.026, $\eta_{\mathrm{group}}^{2}$ = 0.127. Planned contrasts revealed that acquisition of academic writing skills was significantly higher in the group which received combined training with the self-monitoring strategy compared to the group which received combined training with the summarization strategy, *t*(54) = –2.459, *p* = 0.017, $\eta_{\mathrm{group}}^{2}$ = 0.101, as well as compared to the control group, *t*(54) = –2.364, *p* = 0.022, $\eta_{\mathrm{group}}^{2}$ = 0.094. The third variables, prior knowledge of reproduction of text structure knowledge [*F*(1,54) = 0.070, *p* = 0.793], prior knowledge of application of text structure knowledge [*F*(1,54) = 2.575, *p* = 0.114], and prior knowledge of reduction of text content [*F*(1,54) = 3.559, *p* = 0.065] were not significantly related to acquisition of academic writing skills.

This result indicates that combined training of cognitive and metacognitive strategies, in the present case the application of text structure knowledge strategy and self-monitoring strategy, is more effective for the acquisition of academic writing skills than training of only single or two combined cognitive strategies.

In hypothesis 2, we assumed that additional training of a self-monitoring strategy fosters the *text quality* more than additional training of a summarization strategy or no additional training. We assumed that third variables, i.e., *text quality after summarization* and *text quality after revision*, are related to the *text quality of the abstract*.

Our results show a significant difference between the three treatment groups concerning abstract quality, *F*(2,55) = 3.560, *p* = 0.006, $\eta_{\mathrm{group}}^{2}$ = 0.168. Planned contrasts revealed that abstract quality was significantly higher in the group which received combined training with the self-monitoring strategy compared to the group which received combined training with the summarization strategy, *t*(55) = –3.153, *p* = 0.003, $\eta_{\mathrm{group}}^{2}$ = 0.153, as well as compared to the control group, *t*(55) = –2.568, *p* = 0.013, $\eta_{\mathrm{group}}^{2}$ = 0.107. The third variable *text quality after summarization* [*F*(1,55) = 0.053, *p* = 0.819], was not significantly related to text quality of the abstract; however, *text quality after revision* [*F*(1,55) = 14.926, *p* < 0.001, $\eta_{\mathrm{group}}^{2}$ = 0.213] was significantly related to text quality of the abstract.

This result indicates that combined training of cognitive and metacognitive strategies, in the present case the application of text structure knowledge strategy and self-monitoring strategy, is more effective for the abstract quality than training of only single or two combined cognitive strategies. Furthermore, the results underline the influence of text revising for improving text quality.

## Discussion

Academic writing is a challenging task – not only for beginners. In this experimental study, we investigated the effectiveness of the combined training of a cognitive writing strategy and a metacognitive writing strategy, against combined training with two cognitive writing strategies and training of cognitive writing strategy alone on the acquisition of academic writing skills and on abstract quality. To this aim, in the training interventions, we combined a basic cognitive writing strategy concerning text structure with either a cognitive writing strategy on summarization or a metacognitive writing strategy on self-monitoring.

Concerning the *acquisition of academic writing skills*, we found that learners do not benefit more from training with two strategies than from training with one strategy. However, it is not a trivial finding. Although one might assume that two strategies would be more beneficial than one, research on the role of working memory in writing development (McCutchen, 1996; Paz, 2007; Kellogg et al., 2013) indicates that working memory should be dealt with carefully in order to prevent cognitive overload. It is rather the case that the training which combined the cognitive strategy with a metacognitive strategy was most effective for the acquisition of academic writing skills. This result is in line with previous research (Veenman and Beishuizen, 2004).

Concerning the *writing quality*, we found that the group which received additional training on self-monitoring the writing process outperformed the group which received additional training on summarization and the group which did not receive any additional training. We additionally found that the revision process supported with the self-monitoring strategy improved writing quality. The process of revising and evaluating the text written so far was found to be a promising means to enhance text quality (Van den Bergh et al., 1994). In a recent review of research on writing revision MacArthur (2012) could define revision as a problem solving process for detecting discrepancies between actual and intended level of text quality and the consideration of alternatives. The self-monitoring strategy training that we implemented in this study drew attention on these differences as it focused on preparation of the writing process and generating the text in a recursive manner. With prompt cards we supported the detection of the problems which arise while writing and invited for problem correction. Problem detection and problem correction are subprocesses of the revision process (Hayes, 2004). Hence, this finding confirms that revision is important for improving writing quality.

For both research questions we found that learners benefit from the combination of cognitive and metacognitive strategies. Cognitive and metacognitive processes are involved in the writing process (Hayes and Flower, 1980; Hayes, 2012): self-monitoring controls planning, translating, and reviewing the writing process. As metacognitive strategies and cognitive strategies alternate (Pintrich et al., 1993; Boekaerts, 1999), working memory resources do not come into conflict: Writers can switch from cognitive to metacognitive strategies and vice versa. Furthermore, the use of metacognitive strategies facilitates the adaptation of cognitive writing strategies in order to deal with writing deficits (Boekaerts, 1999). Thus, the combination of the two seems to be effective in fostering the academic writing. We could confirm this assumption with our findings concerning both acquisition of writing skills and writing quality.

In general, our results confirm that psychology undergraduates are in need of support in academic writing. First, they need to be prepared to know and apply the text structure of the most important genre in their community, the empirical article. Second, they need to learn, apply, and broaden metacognitive strategies in order to master academic demands in writing. With respect to these two concerns, training of text structure knowledge application in combination with self-monitoring of the writing process showed significant effects in our short-time intervention. From these findings, it can be derived that success would be much greater if such training were to be implemented in the curriculum.

Self-regulated strategy development offers a collection of best teaching practices for teachers to support their students effectively. It provides supportive instruction on how to start writing and how to master each writing step. Furthermore, SRSD supports writers’ interaction, strengthening motivation and self-efficacy (Harris and Graham, in press). These practices have been tested in primary and high schools (Graham et al., 2013). MacArthur et al. (2015) were the first to investigate SRSD at the college level, with a three-year longitudinal study which included strategies for planning, drafting, and revising. With their results – positive effects on writing quality and length of persuasive essays, positive effects on self-efficacy and motivation – they were able to confirm the effectiveness of SRSD also at the college level. Taking together the findings from MacArthur et al.’s (2015) curriculum-embedded face-to-face approach and our short-time computer-based learning approach, we can confirm the need for writing support, and offer tools to master these needs in order to develop writing support effectively.

It might be assumed that if writing courses were offered, students would feel encouraged to develop their writing skills, and would (need to) keep trying to do so if such courses were mandatory. However, research on a variety of methods for organizing and sequencing writing courses at colleges shows that students’ resistance to this kind of support may increase (Adams et al., 2009). In higher education, self-regulation is becoming increasingly crucial for study success. E-learning can provide the opportunity to work on a learning program in an independent and self-regulated manner; therefore, it seems an appropriate means with which to offer writing courses for students in higher education. We hope that our research provides a promising step toward a computer-based approach to writing intervention in higher education.

### Limitations

Our research is limited by several aspects. As writing is a complex process, training can only apply single aspects at a time. Although we investigated promising strategies for writing development in higher education, there are further possible strategies, such as sentence-combining strategies (see Kellogg and Whiteford, 2009). Furthermore, the instruments developed for this study need further refinement. Another limitation is that we did not control the use of the prompt cards. Finally, as the participants were primarily female psychology undergraduates, the generalizability of the results to other groups is limited.

### Future Directions

Further research into the promising idea of combining text structure knowledge application strategies and self-monitoring strategies is needed. Collaboration with peer students is recommended for improving writing skills (Zimmerman and Kitsantas, 2002; Kellogg and Whiteford, 2009; Schriver, 2012) for multiple reasons: As negotiating, questioning, and explaining to learners help them to develop awareness of how to use their strategies effectively, collaboration can support learners in dealing critically with the challenges of the writing task (Englert et al., 2006). Joint regulation between peers can help to reduce the cognitive load of processing, as the load is shared (Topping and Ehly, 1998; Kirschner et al., 2009). Collaboration can positively affect the revision of text products (Van Steendam et al., 2010), and also offers opportunities for perspective-taking to gain an impression of the reader’s needs (Schmitt and Grabowski, 2012). Thus, collaboration with peer students at selected points of the writing process might help novice writers to become aware of how to adapt the self-monitoring strategy to their own and the reader’s needs. Future research should implement this type of training in the curriculum. In a longitudinal study, the writing training can be used to help students to consolidate their writing knowledge and use of writing strategies. Furthermore, a longitudinal study employing repeated writing practice with feedback for revision would also support the acquisition of writing skills, thus fostering writing quality. Over time, students can then elaborate their skills and gain a deeper understanding of which skills should be used, when, and why. Finally, future research should include participants who are more heterogeneous with respect to gender and writing expertise. All recommendations for future research should consider an e-learning approach.

### Conclusion

We were able to show that the writing training interventions applied in our study were effective, even though we provided only a small amount of time. Our findings confirm the importance of applying metacognitive strategies in higher education (Flavell, 1976; Glaser, 1990; Veenman and Beishuizen, 2004). In addition, our findings suggest that combining the cognitive strategy of text structure knowledge application with the metacognitive strategy of self-monitoring supports the development of academic writing in higher education. We believe that our study contributes to the understanding of how combined strategies can work for novice academic writers.

## Author Contributions

The author confirms being the sole contributor of this work and approved it for publication.

## Conflict of Interest Statement

The author declares that the research was conducted in the absence of any commercial or financial relationships that could be construed as a potential conflict of interest.
